# Eficiencia tras la integración de un hospital con *nuevo modelo de gestión*

**DOI:** 10.23938/ASSN.1162

**Published:** 2026-04-29

**Authors:** Mateo Cacho Uzal, Juan Cueva Ares, Fe Lopez-Juiz, Francisco Reyes-Santías

**Affiliations:** 1 Universidad de Santiago de Compostela Santiago de Compostela España; 2 Ministerio de Justicia Tribunal Superior de Justicia de Galicia A Coruña España; 3 Universidad de Vigo Vigo España; 4 CardioCHUS Complejo Hospitalario Universitario de Santiago Santiago de Compostela España; 5 Centro de Investigación Biomédica en Red-Enfermedades Cardiovasculares (CiberCV) Madrid España

**Keywords:** Gestión Sanitaria, Colaboración Público-Privada, Análisis Envolvente de Datos, Eficiencia, Privatización, Health Care Management, Public-Private Sector Partnerships, Data Envelopment Analysis, Efficiency, Privatization

## Abstract

**Fundamento:**

Este estudio compara la eficiencia de un mismo hospital bajo gestión privada (2015-2018) y pública (2019-2022), evaluando el impacto del periodo pandémico (2020-2021).

**Metodología:**

La eficiencia hospitalaria se evaluó mediante un modelo de análisis envolvente de datos, orientado a outputs y con retornos variables a escala. Las consultas, urgencias, intervenciones quirúrgicas, ingresos hospitalarios y estancias se incluyeron como outputs, considerando un input constante (=1). La robustez se analizó con un *bootstrap* paramétrico de 1.500 réplicas, generando intervalos de confianza e indicadores corregidos por sesgo. Además, se aplicó un análisis de series temporales interrumpidas para proyectar un escenario contrafactual del periodo de gestión privada y compararlo con el de gestión pública.

**Resultados:**

La gestión privada alcanzó consistentemente niveles de eficiencia cercanos a la frontera durante 2015-2018. La gestión pública mostró una caída abrupta en 2020-2021, con valores de eficiencia próximos a cero. El análisis *bootstrap* confirmó que estas reducciones fueron estadísticamente significativas y no aleatorias. En 2022 se observó una recuperación parcial, aunque los niveles de eficiencia se mantuvieron por debajo del escenario contrafactual proyectado.

**Conclusiones:**

Mientras la gestión privada mostró una eficiencia hospitalaria estable, la pandemia impactó negativamente la gestión pública, con signos de resiliencia en la etapa pospandémica inmediata. La eficiencia hospitalaria es altamente sensible a las crisis externas y a las estructuras de gobernanza. Fortalecer la resiliencia, la digitalización y la sostenibilidad constituye una estrategia clave para mantener la eficiencia y la equidad en futuras disrupciones sistémicas.

## Abstract (en)

**Background:**

This study evaluates the efficiency of a hospital under two management models, private (2015-2018) and public (2019-2022), and examines the impact of the COVID-19 pandemic (2020-2021).

**Methods:**

Hospital efficiency was evaluated using an output- oriented data envelopment analysis model with variable returns to scale. Outputs included consultations, emergency visits, surgical procedures, admissions, and inpatient stays, assuming a constant input (=1). Robustness was assessed using a parametric bootstrap (1,500 replications) to obtain bias-corrected efficiency scores and confidence intervals. An interrupted time series analysis was conducted to estimate a counterfactual scenario under continued private management and compare it with observed outcomes under public management.

**Results:**

Hospital efficiency under private management remained consistently close to the frontier during 2015-2018. Under public management, efficiency declined sharply during the pandemic, reaching near-zero values in 2020-2021. Bootstrap results confirmed that these reductions were statistically significant and not random. A partial recovery was observed in 2022; however, efficiency remained below counterfactual projections.

**Conclusions:**

While efficiency performed stably under private management, the public model was markedly affected by the pandemic, though showing signs of resilience thereafter. These findings highlight the sensitivity of hospital efficiency to external crises and governance structures, underscoring the need to strengthen resilience, digitalization, and sustainability to maintain efficiency and equity in future health system disruptions.

## INTRODUCCIÓN

Este estudio se sitúa en el debate sobre los modelos de gestión hospitalaria en España, distinguiendo entre gestión directa -realizada con medios propios de la Administración- y gestión indirecta, articulada mediante fórmulas jurídico-organizativas que permiten incorporar a entidades públicas o privadas para la prestación de servicios sanitarios conforme a lo establecido en la Ley 15/1997^1^.

Dentro de esta segunda categoría destacan las colaboraciones público-privadas, cada vez más utilizadas tanto en países desarrollados como en economías emergentes. España constituye un caso de referencia en la aplicación de estos esquemas, especialmente en el ámbito hospitalario, como ilustra la experiencia de la subregión valenciana dentro de la Eurorregión Pirineos Mediterráneo. En ella, el conocido *modelo Alzira* se cimentó sobre un acuerdo concesional por el que la administración autonómica encargaba a entidades privadas la construcción, el equipamiento y la prestación asistencial del hospital a cambio de un pago capitativo anual preestablecido^2^.

Este marco normativo y organizativo ha alimentado un debate político de creciente intensidad en torno a la reversión de servicios privatizados y la recuperación de su gestión directa por parte de las administraciones públicas. Dicho debate plantea cuestiones complejas relacionadas con la integración del personal, la extinción de entidades sujetas a derecho privado y los mecanismos de subrogación, que pueden dar lugar a situaciones de inseguridad jurídica y tensiones laborales cuando el personal pasa a depender de entes públicos sin adquirir plenamente la condición de empleado público^3^. La legislación laboral y administrativa introduce figuras híbridas -como el personal fijo no permanente o la integración sin consolidación de plaza- que permiten la continuidad del empleo pero generan limitaciones en materia de movilidad, estabilidad y consolidación, lo cual ha sido fuente de controversia en diversos procesos de reversión^4^^,^^5^.

Desde el punto de vista académico, la literatura sobre eficiencia hospitalaria ha crecido notablemente, impulsada por la necesidad de maximizar la producción de servicios de salud en contextos de restricciones presupuestarias. Los enfoques no paramétricos, especialmente el análisis envolvente de datos (DEA), se han consolidado como metodologías habituales para medir la eficiencia técnica de los hospitales. Sin embargo, los resultados empíricos no son concluyentes. Algunos estudios previos han mostrado que la eficiencia varía significativamente entre hospitales públicos según factores como tamaño, cartera de servicios y características poblacionales^6^, lo que cuestiona la idea de que la titularidad o el modelo de gestión expliquen de manera directa el desempeño. Más aún, análisis multinivel han identificado diferencias asociadas al tipo de propiedad, pero no homogéneas entre categorías de hospitales, sugiriendo que el marco institucional y organizativo desempeña un papel modulador que impide extraer conclusiones generalizables^7^.

De manera complementaria, investigaciones posteriores han puesto de manifiesto que múltiples variables exógenas -entre ellas factores socioeconómicos, epidemiológicos o territoriales- influyen sobre la eficiencia, incluso más que la estructura de propiedad, tal como revelan modelos avanzados como la regresión beta inflada^8^. Otros autores han llegado a conclusiones similares al analizar la relación entre titularidad y frontera de eficiencia, evidenciando que la propiedad -pública o privada- no constituye por sí sola un determinante robusto del desempeño técnico^9^. En conjunto, la literatura muestra un campo de estudio fragmentado y heterogéneo, marcado por limitaciones metodológicas: predominio de estudios transversales, insuficiente consideración de procesos de transición organizativa y ausencia de análisis en periodos de choque sistémico como la pandemia de COVID-19, evento crítico que alteró profndamente la organización y la actividad de los hospitales, afectando a la eficiencia de forma abrupta. Pese a ello, pocos trabajos han examinado cómo los cambios de gestión interactúan con crisis externas para modificar la trayectoria de eficiencia. La evidencia disponible sugiere que las dinámicas de eficiencia son sensibles a los choques exógenos y que los hospitales enfrentan impactos diferenciados según sus estructuras de gobernanza, sus incentivos y su capacidad de adaptación^10^.

En este contexto emerge el valor del presente estudio, que analiza la eficiencia hospitalaria en dos etapas diferenciadas del mismo centro: gestión privada (2015-2018) y gestión pública (2019-2022), incorporando el efecto disruptivo de la pandemia de COVID-19 como punto de ruptura. Este enfoque permite abordar un vacío de la literatura: evaluar cómo la transición desde un modelo concesional hacia la gestión directa afecta a la eficiencia en un entorno real, y cómo una crisis sanitaria global modifica esa trayectoria.

## MATERIAL Y MÉTODOS

### Diseño del estudio y horizonte temporal

Estudio observacional longitudinal con diseño comparativo antes-después sustentado en series temporales agregadas, realizado en el Hospital Universitario de La Ribera de Alzira (Valencia, España) entre 2015 y 2022.

El periodo analizado permite incorporar tanto la etapa de gestión privada (por concesión) (ALZPRIV, 2015-2018) como la etapa de gestión pública directa (ALZPUB, 2019-2022), iniciada tras la reversión del contrato. Esta división temporal responde al cambio institucional de 2018, considerado un punto de ruptura relevante en el funcionamiento y la gobernanza del hospital. La selección de los años responde a la disponibilidad y homogeneidad de las fuentes de datos.

Cada año incluido en la serie se concibe como una unidad de decisión (DMU), por lo que el hospital se trata como un sistema productivo distinto en cada ejercicio anual. Este enfoque permite evaluar la evolución interanual y comparar trayectorias de eficiencia bajo ambos modelos de gestión en un marco temporal amplio.

### Descripción del modelo concesional y del esquema de financiación

El artículo detalla el sistema de financiación sanitaria utilizado durante el periodo concesional, basado en asignaciones capitativas, mecanismo ampliamente estudiado en la literatura^11^. En estos modelos, el cálculo del presupuesto se realiza por persona asignada a un área sanitaria, con el objetivo de ajustar los recursos al riesgo y a las necesidades de salud de la población. En el caso del Hospital Universitario de La Ribera, este esquema capitativo se combinó con una concesión administrativa donde un consorcio privado asumía actividades de construcción, mantenimiento y prestación asistencial a cambio de una prima per cápita anual abonada por la Generalitat Valenciana^12^.

### Series temporales interrumpidas (ITS): estructura del modelo

Para evaluar el efecto del cambio de gestión sobre la producción hospitalaria, el estudio aplica un diseño de serie temporal interrumpida *(Interrupted Time Series*, ITS). El horizonte temporal analizado (2015-2022) permite identificar claramente dos tendencias: la previa al cambio de gestión (2015-2018), correspondiente al periodo del nuevo modelo de gestión, y la posterior a la intervención (2019-2022), bajo gestión pública. El año 2019 se define como punto de intervención, ya que es el primer ejercicio completo bajo gestión pública directa.

La variable dependiente del modelo ITS es el logaritmo del output total hospitalario, calculado como la suma de consultas externas, urgencias, intervenciones quirúrgicas y altas hospitalarias ajustadas por *case-mix*. Esta aproximación permite homogeneizar unidades de salida y facilita la interpretación en términos de elasticidades.

El modelo se estima mediante una regresión lineal logarítmica que incorpora cambios de nivel, cambios de tendencia, covariables hospitalarias (inputs), e interacciones entre la intervención y las covariables (Anexo):

$$In\left({Output}_{t}\right)=\beta_{0}+\beta_{1}{Time}_{t}+\beta_{2}{Intervention}_{t}+\beta_{3}{TimeAfter}_{t}+\beta_{4}{Trab}_{t}+\beta_{5}G_{pers,t}+\beta_{6}G_{tot,t}+\beta_{7}{Camas}_{t}+\beta_{8}\left({Trab}_{t}\times {Intervention}_{t}\right)+\beta_{9}\left(G_{pers,t}\times {Intervention}_{t}\right)+\beta_{10}\left(G_{tot,t}\times {Intervention}_{t}\right)+\beta_{11}\left({Camas}_{t}\times {Intervention}_{t}\right)+\varepsilon_{t}$$

Dado el tamaño reducido de la serie, el modelo aplica el principio de parsimonia, limitando el número de parámetros para evitar sobreajuste, conforme a las recomendaciones internacionales para series temporales cortas.

La estimación se realiza mediante mínimos cuadrados ordinarios (OLS) con errores estándar robustos para corregir heterocedasticidad y autocorrelación serial (Newey-West, HAC, lag = 1) Se emplea el estadístico de Durbin-Watson para detectar la autocorrelación de primer orden (relación entre residuos consecutivos).

### Definición y operatividad de inputs en la ITS

La selección de inputs se fundamenta en la teoría de la producción hospitalaria, donde la eficiencia resulta de la transformación conjunta de trabajo y capital:

- Capital físico: el número de camas instaladas se utiliza como un proxy del stock de capital, indicador tradicional en análisis de eficiencia hospitalaria^13^^,^^14^. Las camas reflejan tanto infraestructura física como capacidad instalada para la atención continuada.
- Factor trabajo: número total de trabajadores (indicador directo), gasto de personal (medida de la intensidad de uso) y gasto por trabajador (permite examinar la productividad relativa).
- Gasto total anual: medida global de los recursos operativos disponibles, influido por asignaciones capitativas y decisiones presupuestarias anuales.

Estas variables se integran en el modelo ITS como covariables de control, permitiendo estimar los efectos de la intervención de manera independiente del uso de recursos.

### Análisis envolvente de datos (DEA)

La comparación de eficiencia entre ALZPRIV y ALZPUB se realiza mediante un DEA output-oriented con retornos variables a escala (VRS) vía e^*T*^ λ=1. El modelo se basa en una única DMU-hospital por cada año, un input constante (=1) para todas las observaciones, y cinco outputs principales:

- número de consultas totales (consulta: acto médico realizado de forma ambulatoria, para el diagnóstico, tratamiento o seguimiento de un paciente);
- número de urgencias (número de usuarios dados de alta del servicio de urgencias, que no hayan requerido ingreso en la institución ni traslado a otro centro hospitalario ni hayan fallecido en el servicio de urgencias; se incluyen también las altas voluntarias);
- número total de intervenciones quirúrgicas (actos quirúrgicos llevados a cabo en los quirófanos del centro);
- número de altas (número de pacientes ingresados en el centro durante el año para diagnóstico y/o tratamiento en régimen de internado) ajustado por case-mix para evitar que variaciones en la complejidad clínica y en el consumo de recursos de cada paciente distorsionen las comparaciones interanuales;
- número de estancias (1/estancia) (conjunto de pernocta y tiempo que correspondería al suministro de una comida principal, almuerzo o cena)^15^.

Como muestran los trabajos clásicos de eficiencia sanitaria^14^^,^^16^^,^^17^, estos indicadores permiten captar tanto actividad programada como no programada, ofreciendo una visión equilibrada de la producción hospitalaria antes y durante la pandemia.

Este planteamiento elimina la heterogeneidad de inputs y evalúa exclusivamente la capacidad de generar actividad asistencial con una dotación estándar. Los problemas metodológicos asociados a outputs que deben minimizarse (como la estancia media o las listas de espera) se resuelven aplicando la transformación 1/valor, manteniendo la coherencia interna.

El modelo de envolvente (primal) orientado a outputs con retornos variables a escala (VRS) es:

$$\underset{\phi \lambda }{\max}$$

$$\sum_{j=1}^{n}\lambda_{j}y_{j,r}\geq {\phi y}_{o,r^{'}}\;\;\;\;\;\forall r$$

$$\sum_{j=1}^{n}\lambda_{j}\;=1,\;\;\;\;\lambda_{j}\geq 0,\;\;\;\;\;\phi \geq 1$$

donde φ es el factor radial de expansión de outputs, y λ_j_ las ponderaciones que definen la combinación convexa de las DMU eficientes que conforman la frontera. La eficiencia técnica de Farrell orientada a outputs se define como

$$E_{O}=\frac{1}{\hat{\phi_{O}}}$$

La eficiencia técnica (Farrell, output) se reporta como ef ? (0,1], donde ef = 1 indica DMU eficiente (en la frontera) y ef < 1 indica ineficiencia radial. A partir de ef, se derivan dos métricas de distancia: (i) distancia radial a la frontera = (1/ef) − 1, que expresa el incremento proporcional conjunto en los outputs necesario para alcanzar la frontera técnica manteniendo constantes los inputs, y (ii) brecha respecto a la eficiencia plena = 1 − ef, que cuantifica la pérdida relativa de eficiencia en la misma escala de ef.

La ecuación de distancia a la frontera es:

$$D_{O}=1-E_{O}=1-\frac{1}{\hat{\phi_{O}}}$$

donde D_*o*_ mide la ineficiencia relativa de la DMU_*o*_ respecto a la frontera: valores próximos a 0 indican eficiencia plena mientras que valores cercanos a 1 indican una gran distancia a la frontera.

### Bootstrap: fundamentos y aplicación en este estudio

Se ha empleado un bootstrap paramétrico multiplicativo sobre los outputs para obtener un intervalo de confianza (IC) del 95% y corrección por sesgo (inspirado en Simar-Wilson^18^, adaptado a la configuración de datos). Para cada variable de output r=1, …, s se estimó su variabilidad pre-intervención y se modelizó la incertidumbre a través de un proceso estocástico lognormal que preserva la media; con 10.000 iteraciones^19^ (Anexo). El uso de bootstrap en DEA responde a la necesidad de dotar de robustez estadística a estimaciones determinísticas, especialmente en contextos de alta volatilidad, como el generado por la pandemia.

La combinación de un análisis longitudinal mediante ITS con una evaluación de eficiencia relativa a través de DEA responde a la necesidad de capturar efectos dinámicos (tendencias antes/después del cambio de gestión), estimar efectos estructurales, controlar la influencia de inputs clave, y medir eficiencia relativa mediante una frontera de producción empírica. Permite distinguir entre impactos de gestión pública/privada, efectos de recursos, y efectos exógenos como la pandemia.

## RESULTADOS

Los principales outputs asistenciales del Hospital Universitario de La Ribera mostraron un patrón claro: durante ALZPUB, especialmente en los años 2020 y 2021, se observó una reducción significativa de la producción en todas las variables analizadas. En el periodo ALZPRIV los valores medios de actividad asistencial fueron superiores, sin alcanzar significación estadística, lo que indica que el impacto de la COVID-19 no alteró de forma significativa la producción hospitalaria (Tabla 1). Los intervalos de confianza reflejan la mayor estabilidad de los valores en ALZPRIV y la mayor dispersión en ALZPUB, lo que anticipa la ruptura estructural observada durante la pandemia.

**Tabla 1:** Estadísticos descriptivos de actividad asistencial por variable y modelo de gestión

Variable	Hospital Universitario de La Ribera		p*
	Media (DE) IC95%		
	ALZPRIV	ALZPUB	
Consultas	507.722,500 (12.240,32)	437.513,000 (55.351,38)	0,057
	488.245,42-527.199,58	349.436,61-525.589,39	
Urgencias	116.113,250 (62.76,35)	110.443,500 (16.039,50)	1,000
	106.126,18-12.6100,32	84.921,08-135.965,92	
Intervenciones quirúrgicas	21.953,500 (309,51)	18.530,500 (2.850,24)	0,343
	21.461,00-22.446,00	13.995,14-23.065,86	
Altas ajustadas por case-mix	22.741,750 (491,95)	22.282,250 (15.41,69)	1,000
	21.958,95-23.524,55	19.829,07-24.735,43	
Estancias	106.752,750 (36.16,23)	10.466,000 (3.328,36)	0,686
	100.998,53-112.506,97	99.366,84-109.959,16	

ALZPRIV: gestión privada por concesión (2015-2018); ALZPUB: gestión tradicional pública directa (2019-2022); *: U Mann-Whitney.

Los coeficientes del modelo ITS para el logaritmo del output total hospitalario (variable dependiente) mostraron una tendencia ascendente y estadísticamente significativa en la etapa previa (2015-2018), un cambio estructural tras la intervención de 2019, con un descenso marcado en la producción ajustada, y un impacto sustantivo de los recursos hospitalarios sobre la producción (trabajadores, gasto de personal, gasto total y número de camas), aunque con comportamientos diferenciados antes y después del cambio de gestión (Tabla 2).

**Tabla 2:** Coeficientes del modelo de serie temporal interrumpida para la variable dependiente logaritmo del output total hospitalario del Hospital Universitario de La Ribera

Variable	Coeficiente (EE)	IC 95%	p
**Covariables**			
Antes de la Intervención	0,0376 (0,0138)	0,0106 - 0,0647	0,0064
Después de la Intervención	0,0052 (0,0138)	-0,0219 - 0,0323	0,7062
Trabajadores	-0,0004 (0,0002)	-0,0009 - 0,0001	0,0842
Gasto en personal	-0,002 (0,0012)	-0,0043 - 0,0003	0,0842
Gasto total	0,0657 (0,0005)	0,0648 - 0,0666	<0,0001
Camas	-0,094 (0,000)	-0,094 --0,094	<0,0001
**Términos de interacción**			
Trabajadores x Intervención	-0,0017 (0,0002)	-0,0022 - -0,0013	<0,0001
Gasto de personal x Intervención	-0,0083 (0,0012)	-0,0106 - -0,006	<0,0001
Gasto total x Intervención	0,0073 (0,0007)	0,006 - 0,0086	<0,0001
Camas x Intervención	-0,0948 (0,000)	-0,0949 - -0,0948	<0,0001

EE: error estándar robustos Newey-West (HAC, lag=1), IC: intervalo de confianza.

El modelo explica aproximadamente un 96% de la variabilidad de la eficiencia (R² ajustada=0,9604). El estadístico de Durbin-Watson (≈3) indica que no hubo autocorrelación significativa de primer orden en los residuos, lo que respalda la validez del ajuste en un contexto de serie temporal corta. ^Por ejemplo, el gasto total mantuvo un efecto positivo durante todo el periodo, mientras que la influencia de los trabajadores y del gasto de personal perdió intensidad tras la intervención, sugiriendo rigideces o reorientaciones organizativas propias del modelo público. La interacción entre recursos e intervención indica que el cambio de modelo de gestión modificó la elasticidad con la que los inputs afectan a los outputs.

**Figura 1:**
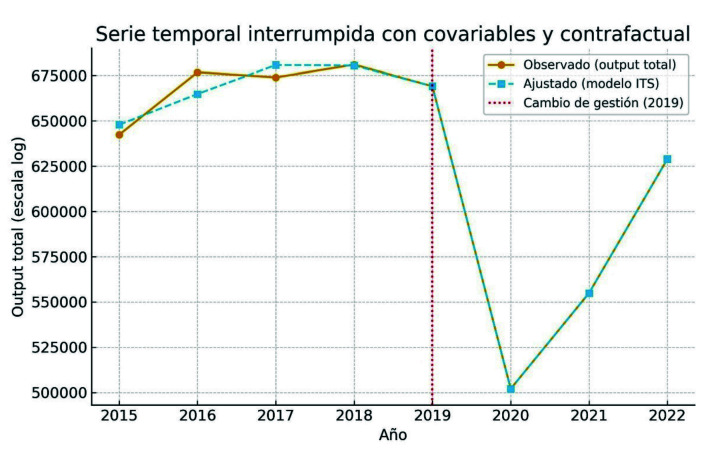
Eficiencia hospitalaria en serie temporal interrumpida ITS con covariables e interacciones: comparación entre las etapas de gestión por concesión (ALZPRIV) y pública (ALZPUB) del Hospital Universitario de La Ribera con covariables y escenario contrafactual (2015-2022). La línea vertical indica el inicio del primer año completo con gestión pública.

La figura 1 ilustra gráficamente la trayectoria observada del output total, la tendencia estimada por el modelo, la línea contrafactual estimada para ALZPRIV y la ruptura de la serie a partir de 2019. La caída más acusada aparece en 2020-2021, coincidiendo con la pandemia, en la que el output total se situó muy por debajo del contrafactual. En 2022 se observa una recuperación parcial, pero sin retornar a la trayectoria previa.

**Tabla 3:** Eficiencia técnica obtenida mediante análisis envolvente de datos, bajo orientación output y retornos variables a escala (VRS), con intervalos de confianza obtenidos por bootstrap

Gestión	Año	Eficiencia	Distancia radial	Brecha	IC 95%
		ef	1/ef-1	1-ef	
ALZPRIV	2015	1,000	0,000	0,000	1,000-1,000
	2016	1,000	0,000	0,000	1,000-1,000
	2017	1,000	0,000	0,000	1,000-1,000
	2018	1,000	0,000	0,000	1,000-1,000
ALZPUB	2019	1,000	0,000	0,000	1,000-1,000
	2020	0,859	0,164	0,141	0,859-0,886
	2021	0,907	0,102	0,093	0,907-0,933
	2022	1,000	0,000	0,000	1,000-1,000

ALZPRIV: gestión privada por concesión (2015-2018); ALZPUB: gestión tradicional pública directa (2019-2022); Distancia radial: indica el incremento proporcional conjunto en los outputs necesario para alcanzar la frontera técnica manteniendo constantes los inputs; Brecha: representa la pérdida relativa de eficiencia en la misma escala de la eficiencia técnica; IC95%: intervalo de confianza percentílicos basado en bootstrap (10.000 réplicas).

La tabla 3 muestra las eficiencias técnicas para los años 2015-2022, junto con sus intervalos de confianza, la distancia radial a la frontera y la brecha de eficiencia.

Estos resultados permiten diferenciar claramente las dos etapas:

- Etapa ALZPRIV (2015-2018) caracterizada por 1) eficiencias exactamente iguales a 1, indicando desempeño pleno en la frontera eficiente; 2) distancias radiales y brechas iguales a 0, confirmando rendimiento óptimo para todos los años del periodo, y 3) intervalos de confianza estrechos que muestran estabilidad y robustez.
- Etapa ALZPUB (2019-2022) caracterizada por eficiencias variables: en 2019 eficiencia aún igual a 1, reflejando continuidad inicial del desempeño; en 2020 eficiencia de 0,859, con una distancia radial significativa (0,164); en 2021, eficiencia de 0,907, mostrando una leve recuperación, pero aún lejos de la frontera; en 2022 la eficiencia vuelve a 1, lo que sugiere una recuperación del nivel de producción relativo, aunque en un contexto asistencial distinto al previo a la pandemia. Los intervalos bootstrap confirman que las reducciones observadas en 2020-2021 fueron estadísticamente significativas, descartando explicaciones basadas en variabilidad aleatoria.

**Figura 2:**
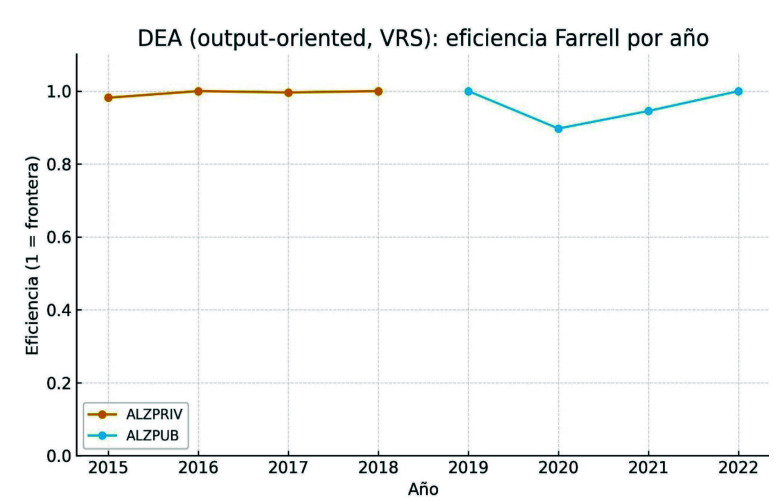
Distribución de las puntuaciones de eficiencia técnica obtenidas mediante análisis envolvente de datos, bajo orientación output y retornos variables a escala (VRS). El año 2018 representa la transición de modelo, con meses de nuevo modelo de gestión y meses de gestión pública directa integrada en el Servicio Valenciano de Salud.

La figura 2 muestra la serie temporal completa de eficiencia DEA, con un patrón altamente estable y eficiente durante ALZPRIV, cercano a la frontera de eficiencia, y puntuaciones más dispersas bajo gestión pública (ALZPUB), con una ruptura abrupta en 2020 y una recuperación parcial y desigual en 2021-2022.

**Figura 3:**
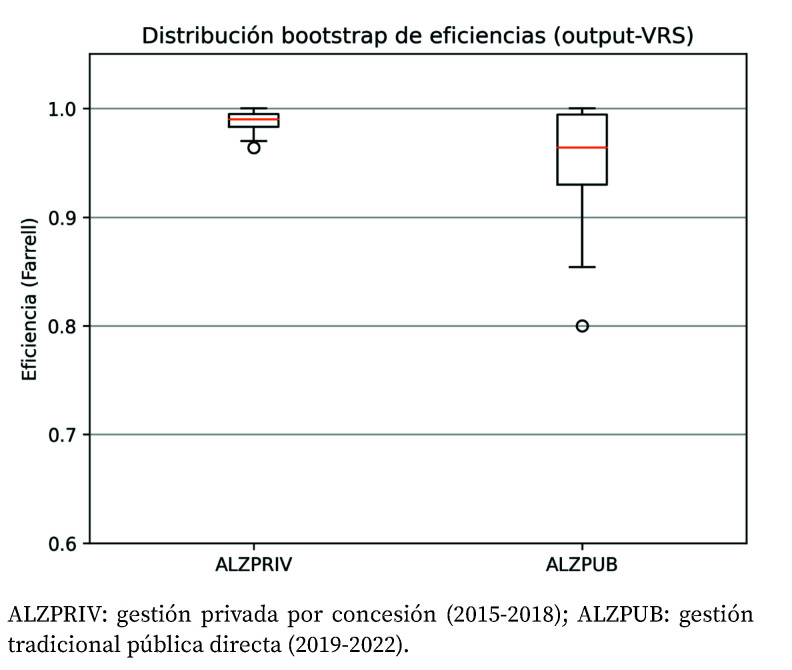
Distribución bootstrap de las puntuaciones de eficiencia hospitalaria, bajo orientación output y retornos variables a escala (VRS). La curva de densidad permite visualizar la variabilidad de las estimaciones y los intervalos de confianza asociados.

ALZPRIV: gestión privada por concesión (2015-2018); ALZPUB: gestión tradicional pública directa (2019-2022).

La figura 3 presenta la distribución de las puntuaciones de eficiencia técnica estimada mediante 10.000 iteraciones de bootstrap aplicadas al análisis envolvente de datos (DEA) con orientación output y retornos variables a escala. El Hospital Universitario de La Ribera bajo gestión privada presenta concentración de eficiencias en torno a la frontera de eficiencia. Sin embargo, bajo gestión pública, la mayor dispersión de las eficiencias indica menor consistencia en el desempeño, y el desplazamiento hacia valores inferiores en 2020 y 2021 sería reflejo de la volatilidad y disrupción producidas por la pandemia.

## DISCUSIÓN

La actividad asistencial del Hospital Universitario de La Ribera mostró un desempeño en frontera altamente estable en la etapa de gestión por concesión, disminuyó durante la etapa de gestión pública, y la eficiencia cayó drásticamente en 2020-2021. Aunque la reversión explicaría cambios organizativos y de tendencia, este colapso de la eficiencia sería atribuible principalmente al shock exógeno de la pandemia de COVID-19. El modelo de ITS reveló que los inputs no afectaban de igual forma antes y después de la reversión, ya que se observó una reducción clara de la elasticidad de los recursos laborales bajo la etapa pública, probablemente por ajustes organizativos y prioridades asistenciales no reflejados en outputs tradicionales. El retorno a valores de eficiencia iguales a 1 en 2022 muestra cierta resiliencia organizativa pero no implica un restablecimiento total del modelo previo, sino un reequilibrio tras la fase aguda de la pandemia.

Estos resultados permiten comprender mejor cómo la transición entre modelos de gestión y la irrupción de un evento disruptivo como la pandemia de COVID-19 pueden modificar de forma sustantiva la eficiencia de un hospital. La comparación entre la etapa concesional y la etapa pública del Hospital Universitario de La Ribera revela patrones diferenciados que deben interpretarse en un marco conceptual amplio, donde intervienen tanto dinámicas institucionales como condicionantes externos y cambios organizativos asociados al modelo de gobernanza.

Los hallazgos muestran que, durante la etapa concesional, el hospital mantuvo niveles de eficiencia técnica muy elevados y estables. La producción asistencial se alineó estrechamente con la disponibilidad de recursos, lo que sugiere una estructura organizativa capaz de transformar de manera eficiente los inputs en outputs. Este comportamiento coincide con trabajos previos^9^^,^^11^ que señalan que las concesiones sanitarias pueden generar altos niveles de eficiencia en contextos donde los incentivos están claramente definidos y los marcos contractuales establecen responsabilidades precisas sobre coste y producción.

Sin embargo, la reversión del modelo en 2018 introduce un marco institucional distinto, en el que la gobernanza pública incorpora objetivos adicionales -como la equidad territorial, la estabilidad laboral o la integración orgánica en el sistema autonómico- que no siempre se traducen en maximización inmediata de actividad. La pérdida de elasticidad entre recursos y producción observada en el periodo de gestión pública puede entenderse en este contexto: la reorganización del personal, la alineación con estructuras retributivas y normativas del sector público, y la redefinición de prioridades asistenciales pudieron generar un periodo de adaptación y reajuste organizativo. Las transiciones de modelo, incluso cuando se acompañan de incrementos presupuestarios, pueden implicar fases de pérdida temporal de eficiencia asociadas a cambios de gobernanza y reestructuración operativa^12^.

Aunque la eficiencia en la etapa concesional fue elevada, esta superioridad debe interpretarse con cautela. La eficiencia técnica no es un constructo aislado del entorno económico y del comportamiento organizativo. En modelos concesionales, determinados incentivos pueden promover estrategias que aumentan la producción o reducen costes de forma significativa, pero que no necesariamente reflejan mejoras estructurales de eficiencia. Por ejemplo, el aumento extraordinario del número de partos durante la etapa concesional, muy por encima del crecimiento experimentado por los hospitales públicos valencianos, permitió aumentar ingresos y reducir el coste medio por episodio. Asimismo, los menores costes salariales y la estructura flexible de contratación típica de las concesiones pudieron contribuir a mejorar indicadores técnicos. Estos aspectos, presentes en las asociaciones público-privadas, muestran que la eficiencia observada puede responder tanto a buena gestión como a comportamientos propios de los sistemas capitativos orientados a maximizar actividad^20^^,^^21^. Además, se han detectado patrones de selección de pacientes en sistemas mixtos, por ejemplo en Australia, donde hospitales privados derivan pacientes complejos o costosos hacia el sector público^20^. Si bien no existen evidencias directas de que esta práctica ocurra en el Hospital Universitario de La Ribera, constituye una advertencia metodológica relevante: cuando la eficiencia se interpreta únicamente desde volúmenes y costes agregados, existe el riesgo de atribuir ventajas estructurales a lo que pueden ser diferencias en composición de casos.

La literatura no ofrece consenso sobre la superioridad de un modelo de gestión respecto a otro. Algunos estudios han mostrado que hospitales de gestión privada o concesional presentan mejores resultados en términos de tiempos de espera, calidad percibida y eficiencia técnica^9^^,^^11^. Otros, sin embargo, apuntan a efectos adversos en recursos críticos, señalando que la presión para reducir costes en modelos privados ha llevado en ciertos contextos a disminuir el número de camas y personal, con impactos negativos en la capacidad de respuesta asistencial a largo plazo^22^.

Asimismo, existen ejemplos de modelos alternativos al binomio público-privado, como las fundaciones públicas, que han demostrado niveles de eficiencia comparables o superiores mediante autonomía de gestión y mecanismos de incentivación interna^20^^,^^22^. Esta heterogeneidad evidencia que la propiedad no determina por sí sola los resultados: lo que importa son los marcos de gobernanza, los incentivos contractuales, el grado de autonomía y la articulación entre recursos y objetivos estratégicos.

En el caso del modelo capitativo valenciano, algunos informes han defendido que el coste para la administración fue significativamente inferior al de la gestión pública, pero la evidencia oficial apunta a una diferencia más moderada^23^^,^^24^. Ello recuerda que las evaluaciones económicas de las concesiones dependen en gran medida de la transparencia de la información y de la metodología empleada para comparar costes.

Si bien la reversión explica parte de la caída abrupta de eficiencia en 2020 y 2021, esta coincide con los efectos de la pandemia de COVID-19 descritos por la literatura internacional: suspensión de actividad programada, sobrecarga asistencial, reorganización interna y prioridades clínicas desplazadas hacia la atención urgente y crítica^25^^-^^28^. Por tanto, la pandemia constituye el elemento más decisivo en la trayectoria observada. Estos resultados se alinean con los de estudios multicéntricos europeos que han documentado retrocesos drásticos en la eficiencia hospitalaria durante la primera fase de la pandemia, identificando pérdidas de productividad técnica y disminución de la capacidad operativa, incluso en organizaciones con buen desempeño previo^26^^,^^27^. Las técnicas bootstrap utilizadas confirman, además, que la reducción observada no es fruto de fluctuación aleatoria, sino un efecto estadísticamente robusto^18^^,^^29^.

La recuperación parcial observada en 2022 refleja la capacidad adaptativa del hospital bajo gestión pública. Investigaciones recientes sugieren que la resiliencia de los centros sanitarios tras la pandemia depende de factores como la digitalización, la flexibilidad organizativa y la capacidad de redefinir procesos asistenciales^30^. La política sanitaria debería incorporar la resiliencia en sus diferentes modelos, porque la eficiencia técnica en condiciones normales no garantiza la capacidad para responder a crisis sistémicas.

El estudio presenta algunas limitaciones que conviene considerar al interpretar los resultados. En primer lugar, el horizonte temporal, aunque adecuado para captar el cambio de modelo de gestión y el impacto de la pandemia, es relativamente reducido para evaluar dinámicas estructurales de largo plazo. Desde el punto de vista metodológico, la medición de la eficiencia se centra en outputs cuantitativos, sin incorporar dimensiones relevantes como la calidad asistencial, los resultados clínicos o la satisfacción de pacientes y profesionales. Finalmente, la coincidencia temporal entre el cambio de gestión y el shock exógeno de la COVID-19 dificulta aislar completamente el efecto atribuible a cada factor.

El enfoque mixto empleado (combinación de ITS con DEA y técnicas de bootstrap) permite obtener una visión sólida y complementaria del comportamiento del hospital al transitar entre modelos de gestión, permitiendo medir el impacto de crisis sistémicas. Pero también arroja luz sobre los límites inherentes a los estudios agregados: la eficiencia hospitalaria no solo depende de inputs y outputs cuantificables, sino de aspectos cualitativos como la calidad asistencial, los resultados clínicos, la satisfacción profesional o la coordinación clínica. La ausencia de estos indicadores en el análisis invita a interpretar los resultados con prudencia y orienta futuras líneas de investigación hacia modelos DEA de redes, análisis de productividad total de los factores o técnicas híbridas que integren calidad y actividad.

En conclusión, la eficiencia hospitalaria del Hospital Universitario de La Ribera experimentó una evolución diferenciada entre las etapas de gestión por concesión y pública: la etapa concesional mostró estabilidad y eficiencia elevada, la etapa pública evidenció vulnerabilidad ante un choque extraordinario, y la recuperación parcial en 2022 apunta a la importancia de la adaptabilidad. Esta variación no puede atribuirse exclusivamente al tipo de gestión, sino a la interacción entre factores institucionales, organizativos y externos.

El caso del Hospital Universitario de La Ribera ofrece un precedente relevante tanto para orientar decisiones futuras en el Sistema Nacional de Salud -teniendo en cuenta que la eficiencia hospitalaria es un fenómeno multidimensional y contextual, dependiente de incentivos, estructuras de gobernanza y capacidad de adaptación-, como para abrir líneas de investigación centradas en calidad, sostenibilidad y resiliencia organizativa. Ningún modelo de gestión asegura la eficiencia en todos los escenarios; lo determinante es cómo cada organización articula sus recursos, incentivos y capacidad de respuesta ante entornos cambiantes.

## Figures and Tables

**Figura A-1:**
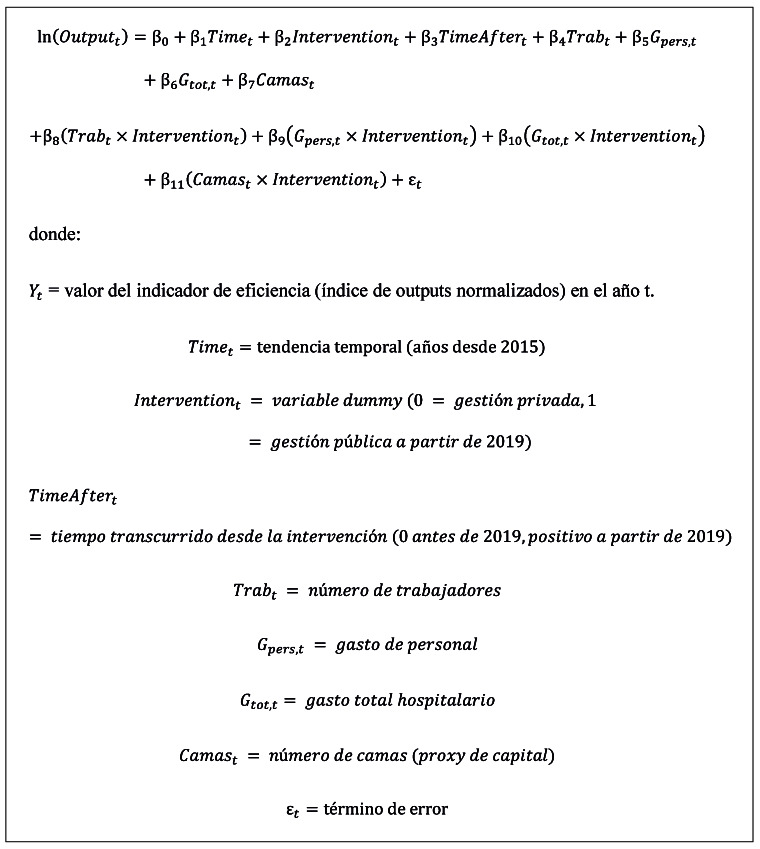
Estimación del modelo de series temporales interrumpidas mediante regresión lineal logarítmica.

**Figura A-2:**
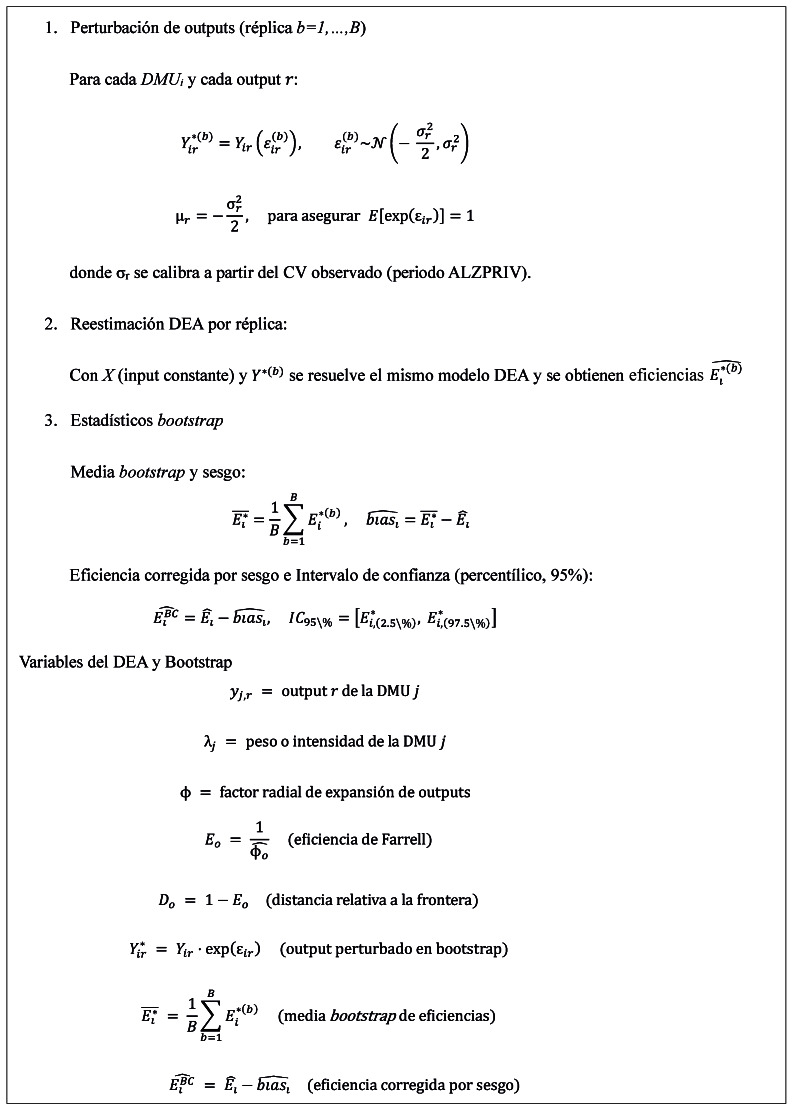
Modelización de la eficiencia mediante 10.000 iteraciones de bootstrap aplicadas al análisis envolvente de datos.

## Data Availability

Se encuentran disponibles bajo petición al autor de correspondencia.
